# Establishment and evaluation of a method for measuring ornithine transcarbamylase activity in micro blood of neonates

**DOI:** 10.1186/s13023-025-03529-2

**Published:** 2025-01-08

**Authors:** Zhilei Zhang, Xin Wang, Jingjing Zhang, Xianwei Guan, Yanyun Wang, Dongyang Hong, Yahong Li, Peiying Yang, Yun Sun, Tao Jiang

**Affiliations:** Nanjing Women and Children’s Healthcare Hospital, Center of Genetic Medicine, The Affiliated Obstetrics and Gynecology Hospital With Nanjing Medical University, No.123, Tianfei Xiang, Mochou Road, Nanjing, Jiangsu China

**Keywords:** Ornithine transcarbamylase deficiency, Ornithine transcarbamylase activity, Newborns, Micro blood, Tandem mass spectrometry, Citrulline

## Abstract

**Background:**

Ornithine transcarbamylase deficiency exhibits a high degree of clinical heterogeneity, making its screening and classification challenging in some instances. In this study, we first established a simple and stable method for testing ornithine transcarbamylase activity using micro blood from newborns, rather than relying on venous blood.

**Methods:**

The activity of ornithine transcarbamylase was assessed by measuring the concentration of citrulline produced in the reaction with carbamoyl phosphate and ornithine, using serum, plasma or micro blood. Correlation analysis was evaluated using Sangerbox Tools. The Receiver Operating Characteristic curve was used in SPSS Statistics 17.0 to evaluate the diagnostic efficiency of Ornithine transcarbamylase deficiency.

**Results:**

A strong linear relationship was observed between ornithine transcarbamylase activity and both micro blood volume and reaction time (R^2^ = 0.9793, 0.9922 respectively). The intra-coefficient variation and inter-coefficient variation were 11% and 12.5% with a 1-h reaction time, and 6.77% and 9.58% with a 3-h reaction time, respectively. And the inter-coefficient variation was lower than the most widely used colorimetry method (5.1–21.1%). The Limit of Blank was 0.57 nmol/mL/h. The reference interval for normal newborn population is greater than or equal to 39.6 nmol/mL/h. Notably, the method exhibited a 100% sensitivity, surpassing the sensitivity of colorimetry method (94.3%), along with and a specificity of 96.9% for diagnosing ornithine transcarbamylase deficiency.

**Conclusions:**

We pioneered a method for testing OTC activity that normally carried on venous blood can be effectively performed on microblood heel samples. Meanwhile, our method presents a simpler, more stable and reproducible approach compared to colorimetry.

## Introduction

Ornithine transcarbamylase deficiency (OTCD, OMIM#300,461) is an X-linked metabolic disorder, characterized by a spectrum of symptoms including neurobehavioral changes, recurrent vomiting, seizures, and other manifestations triggered by high blood ammonia.[1],[2] The clinical phenotype of this disease varies from neonatal to late onset, with neonatal-onset cases exhibiting more severe symptoms and extremely higher mortality. [3] Additionally, some female carriers may manifest clinical phenotypes similar to those observed in male patients. [4] This condition is caused by mutations in the gene coding the mitochondrial enzyme ornithine transcarbamylase (OTC, OMIM#311,250). [5] OTC is primarily located in the mitochondrial matrix, with a portion bound to the inner membrane, and plays a crucial role in urea cycle. [6] This enzyme catalyzes the conversion of ornithine (ORN) and carbamoyl phosphate into citrulline (Cit). [7] OTCD is the most commom urea cycle disorder (UCDs), comprising approximately 50% [8] of cases, with a prevalence of 1/17 000–77,0005 world-wide. [3], [9], [10] Presently, the radical treatment for OTCD remains a liver transplant. [11] However, it's crucial to note that liver transplant cannot reverse nervous system damage induced by hyperammonemia. Wakiya and colleagues [12] recommend that performing liver transplant when the metabolic state stabilizes and significant nervous system damage is absent. Hence, early diagnosis and prompt treatment of OTCD carry immense significance in preventing severe complications.

In neonatal screening, Cit serves as the primary indicator for the initial screening of OTCD. Nevertheless, its reliability is insufficient for OTCD. For instance, an expanded newborn screening program in Tuscany revealed a case of OTCD where the initial screening showed a low Cit level, which subsequently normalized in the follow-up screening. [13] A prior study documented two cases of late-onset OTCD patients exhibiting normal blood citrulline concentrations during newborn screening. [3] And T. Rohininath [14] reported a case of late-onset OTCD with fatal presentation, despite normal cit levels at onset. Thus, as a neonatal screening indicator for OTCD, Cit may present challenges and lead to missed detection. Additionally, a low Cit level still stems from various factors, including malnutrition, [15] related diseases,[16–20] intestinal malrotation [13] or individuals carrying these diseases, as well as some unknown causes. This diversity of factors contributes to a heightened rate of false positives, presenting challenges in clinical practices.

Presently, confirming OTCD in clinic depends primarily on genetic diagnosis or testing OTC enzyme activity, [21] both of which require venous blood collection. This procedure frequently triggers parental anxiety and could result in the reluctance to proceed with further diagnostic steps, especially in patients who do not exhibit clinical and biochemical phenotype. As the gold standard of genetics disease, enzyme activity testing is sufficiently considered for OTCD diagnosis. Many studies have utilized OTC activity testing in clinical settings using plasma or serum. [22] However, within the realm of newborn screening, challenges in obtaining plasma or serum samples from neonatus have constrained the broader application of OTC activity testing in diagnosing OTCD.

Micro blood sampling has proven to be a practical and cost-efficient method across various fields, including antibody testing [23] toxicokinetics, [24] and other clinical chemistry indicators. In this article, we explore a novel approach for applying OTC activity testing in newborns using heel-prick blood samples—a method not previously reported. This method is integrated into the first recall process, conducted simultaneously with biochemical examination. On one hand, if both biochemical indicators and OTC enzyme activity are normal, it can reduce the risk of missed detection. Conversely, if the biochemical indicators are normal but the OTC enzyme activity is reduced, or if both are abnormal, it warrants further diagnostic investigation. This application can decrease missed detection and false positives. On the other hand, it facilitates same-day result reporting enabling swift and timely diagnosis.

## Material and methods

### Samples

Blood samples were collected from the heels of neonates (including 3 individuals with *OTC* gene mutations and 64 healthy controls) into heparin sodium-containing collection tubes, and the OTC activity was measured on the same day. The samples short-stored at 4 ℃. Additionally, venous blood was collected from volunteers into tubes both with and without heparin sodium for extracting serum and plasma, respectively.

Sample collection and detection were approved by the Ethics Committee of Nanjing Maternity and Child Health Care Hospital, affiliated with Nanjing Medical University, China. Informed consent was obtained from the patients or their guardians.

The human studies were conducted following the ethical standards of the institutional and/or national research committee (Nanjing Women and Children's Healthcare Hospital + [2014]36) and the 2013 Helsinki Declaration, including its subsequent amendments or comparable ethical standards.

### The method of OTC enzyme activity testing

Different volumes of samples were added to 0.4 mol/L TEA-HCl (V900257, sigma, USA) (PH = 8.0) containing 2.5 mmol/L ORN (W419001, sigma, USA) and 10 mmol/L carbamoyl phosphate (C5625, sigma, USA) and then incubated at 37 ℃ in a water bath. After reaction period, the process was stopped by 3% sulfosalicylate acid (390,275, sigma, USA). The concentration of Cit, a production of this reaction, was determined by tandem mass spectrometry (MS/MS) after fixation in dried blood spots using NeoBase™ 2 Kit (SY44014-D, PE, USA).

### Homemade quality control products

To monitor the stability of the entire experimental process, we gathered venous blood samples from volunteers, which were then centrifuged them at 3000 rpm for 10 min to separate the components.

Subsequently, we carefully extracted the supernatant, the clear serum atop the centrifuged blood. Finally, the serum was divided them into smaller aliquots and stored at −80℃ to prevent repeated freezing and thawing, preserving its degradation.

### Intra-coefficient variations

We employed self-produced quality control products with 20 repetitions to assess intra-coefficient variations (CV) and evaluate the stability of the experimental procedures. The coefficient of variation was computed by dividing the standard deviation by the average across the 20 repetitions. Outliers were defined as values falling outside the range of mean ± 4 standard deviations (SD).

### Inter-coefficient variations

We utilized self-produced quality control products to evaluate inter-CV, aiming to monitor the accuracy of our experimental procedures. The experiment was repeated four times, with each batch consisting of 20 repetitions. Outliers were defined as values falling outside the range of mean ± 4 SD. The CV was computed by dividing the standard deviation by the average across the 80 repetitions after rejecting discrete values.

### Limit of blank

As described in Clinical and Laboratory Standards Institutehe, [25] limit of Blank (LOB) was calculated by analyzing multiple replicates of the OTC activity using a blank sample following the formula: LOB = mean + 1.645 * SD. LOB represents the highest measurable result expected when repeatedly examining blank samples. The multiplicative factor of 1.645 is based on the assumption of a Gaussian distribution and a 5% false-positive detection rate.

### The diagnostic criteria for OTCD

The confirmation of OTCD in an individual requires suggestive clinical and biochemical findings (such as elevated ammonia, elevated glutamine, and low-to-normal citrulline levels), the absence of another inborn error of metabolism, and one of the following:[26]

1. Molecular genetic testing: A pathogenic (or likely pathogenic) variant in the *OTC* gene.
2. Urine organic acid testing: Increased orotic acid excretion may also show elevated uracil levels after an allopurinol test or in a random urine sample.
3. OTC enzyme activity testing: A decreased OTC enzyme activity in liver.

However, plasma OTC activity can also effectively diagnose OTCD in hemizygous males and in most symptomatic heterozygous females, as outlined in the European consensus guidelines.[16]

### Statistical analysis

Prism Ver. 6.01 (GraphPad) and SPSS 17.0 (IBM) were employed to analyse the data. Mann–Whitney U test was applied to compare skewed distribution data between two groups. *p* < 0.05 was considered statistically significant. In this study, we corrected the skewed data to a normal distribution using a square root transformation and then analyzed this data using Student’s *t*-test to increase the reliability of *p*-value, which was calculated with only 2 positive samples. The Receiver Operating Characteristic (ROC) curve was used in SPSS Statistics 17.0 to evaluate the diagnostic efficiency of OTCD. Correlation analysis was evaluated using Sangerbox Tools (http://www.sangerbox.com/tool).

## Results

### The activity of OTC in serum and plasma

**Fig. 1:**
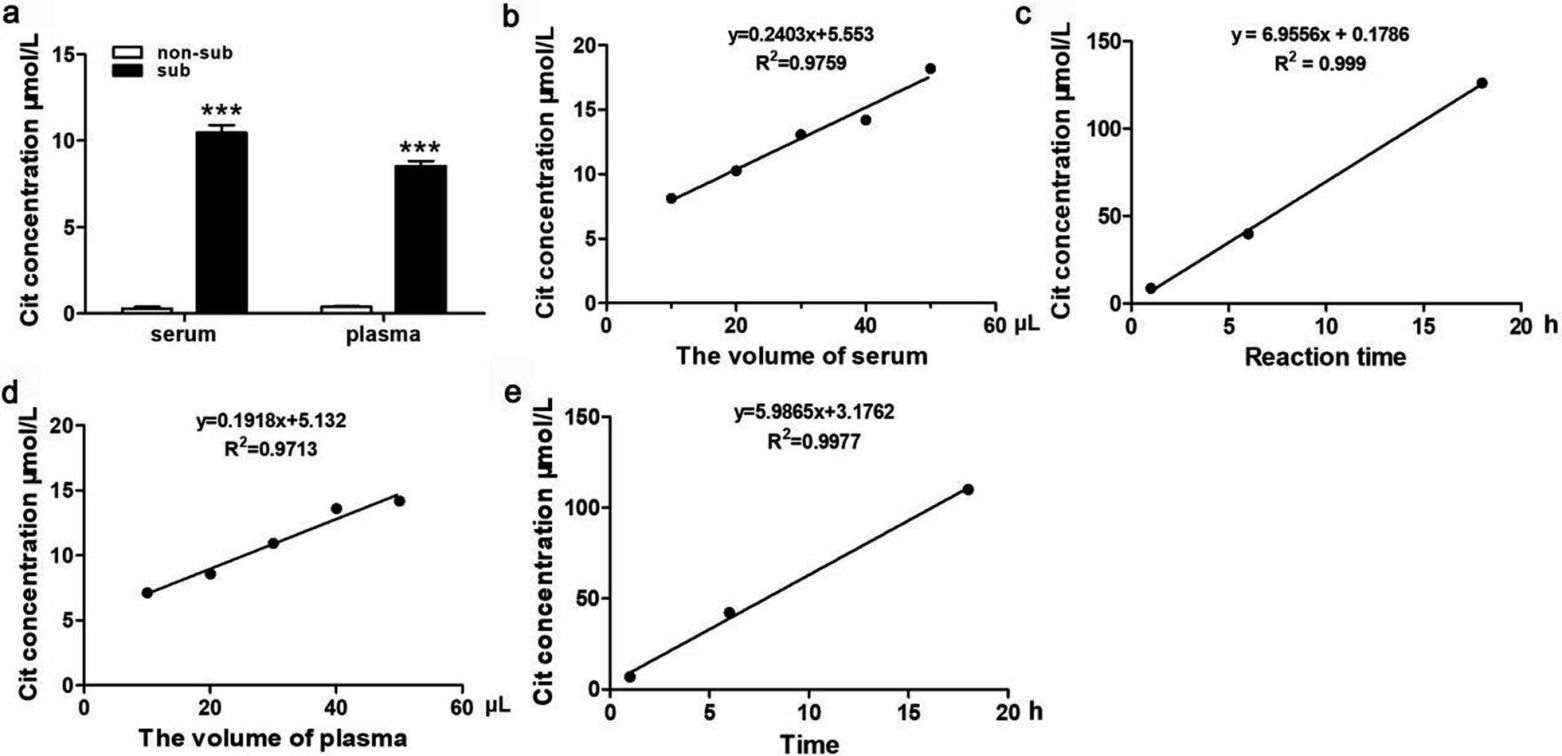
The activity of OTC in serum and plasma. **a** The concentration of Cit was measured after a 1-h enzyme activity reaction using 20 μL of serum or plasma with or without substrates, *** *P* < 0.001, two-tailed. **b**, **d** The concentration of Cit was measured after a 1-h enzyme activity reaction using varying volumes (10, 20, 30, 40, 50 μL) of serum or plasm. **c**, **e** The concentration of Cit was measured at intervals of 1, 6, 18 h following an enzyme activity reaction using 10 μL of serum or plasma

To authenticate the reliability of our OTC activity detection method, we assessed this specific enzyme activity in both serum and plasma, following the previously documented approach. And the results were consistent with previous reported (Fig. 1a). Additionally, our findings reveal a significant increase in Cit production when a substrate was present compared to its absence. This suggests that non-enzymatic Cit production does not influent this reaction system. Notably, our investigation revealed a direct correlation between the product concentration and varying enzyme levels as well as reaction durations. This correlation exhibited a robust linear relationship, with R^2^ values of 0.9759, 0.999, 0.9713, and 0.9971, respectively (Fig. 1b-1e). These findings substantiate the reliability of our enzyme activity detection system.

### Performance verification of OTC activity in micro blood

#### The linearity

**Fig. 2:**
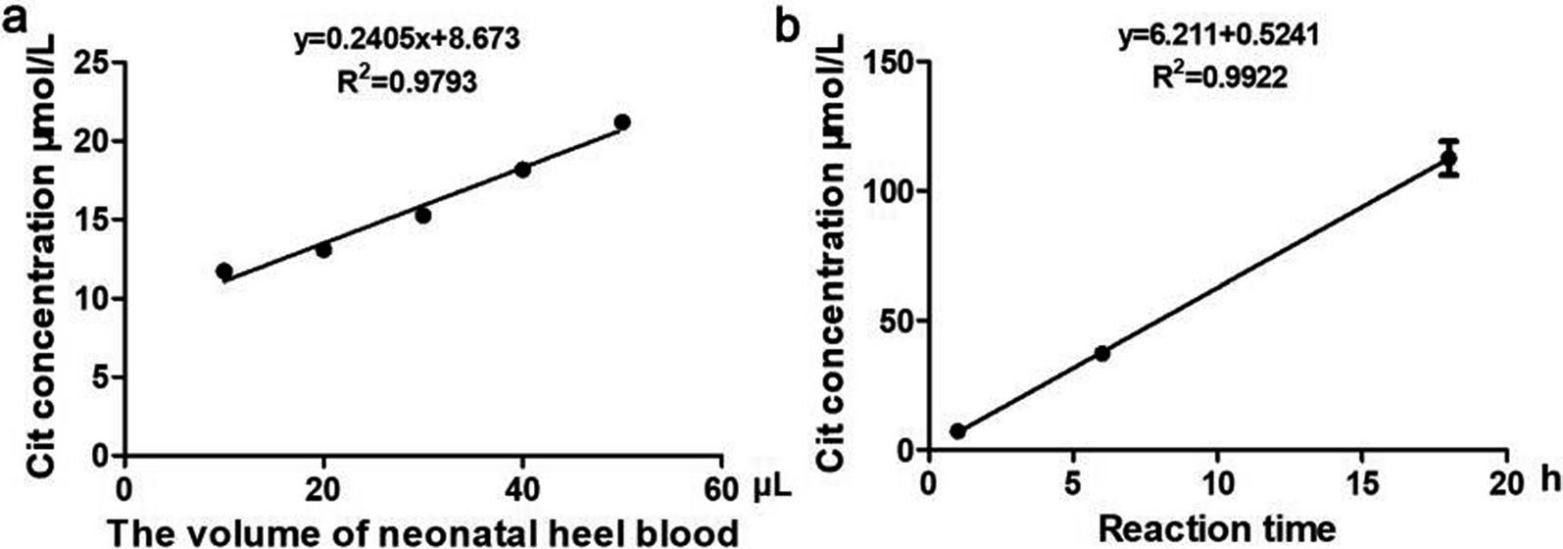
The activity of OTC in micro blood from neonatal heel. **a** The concentration of Cit was measured after a 1-h enzyme activity reaction using varying volumes (10, 20, 30, 40, 50 μL) of micro blood from same neonatal heel. **b** The concentration of Cit was measured at intervals of 1, 6, 18 h following an enzyme activity reaction using 10 μL of micro blood

We further assessed the feasibility of the aforementioned method using micro blood from newborn heel, which more suitable for neonatal screening. And the results indicate a strong and consistent linear relationship between enzyme activity and both sample volume and reaction time, as indicated by the high R^2^ values of 0.9793 and 0.9922, respectively (Fig. 2a, b).

#### Accuracy evaluation

**Fig. 3:**
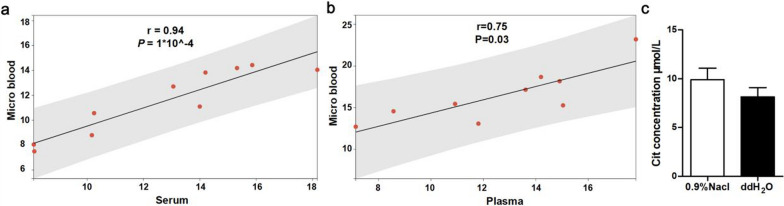
Accuracy evaluation of the OTC activity testing system. **a** The correlation between serum OTC activity and micro blood OTC activity,r = 0.94, P = 1*10^−4, two-tailed, n = 10. **b** The correlation between plasma OTC activity and micro blood OTC activity,r = 0.75, *P* = 0.03, two-tailed, *n* = 10. **c** The concentration of Cit was measured in in micro blood samples diluted with 0.9% NaCl and ddH_2_O, respectively

We assessed the accuracy of OTC activity measurement using micro blood from the same volunteer by comparing it to OTC activity measured in serum or plasma samples. The findings indicated a strong positive correlation between the OTC activity in micro blood and in both serum (r = 0.94, p = 1*10^−4, Fig. 3a) and plasma (r = 0.75, p = 0.03, Fig. 3b). Additionally, to account for the potential influence of hemolysis during the heel blood collection process, we divided the same venous blood sample into two portions. One was mixed with physiological saline, and the other was mixed with an equal volume of double distilled water (ddH_2_O) to induce hemolysis. We tested the OTC activity in both samples and results showed hemolysis almost had no impact on enzyme activity (Fig. 3c).

#### Imprecision evaluation

**Fig. 4:**
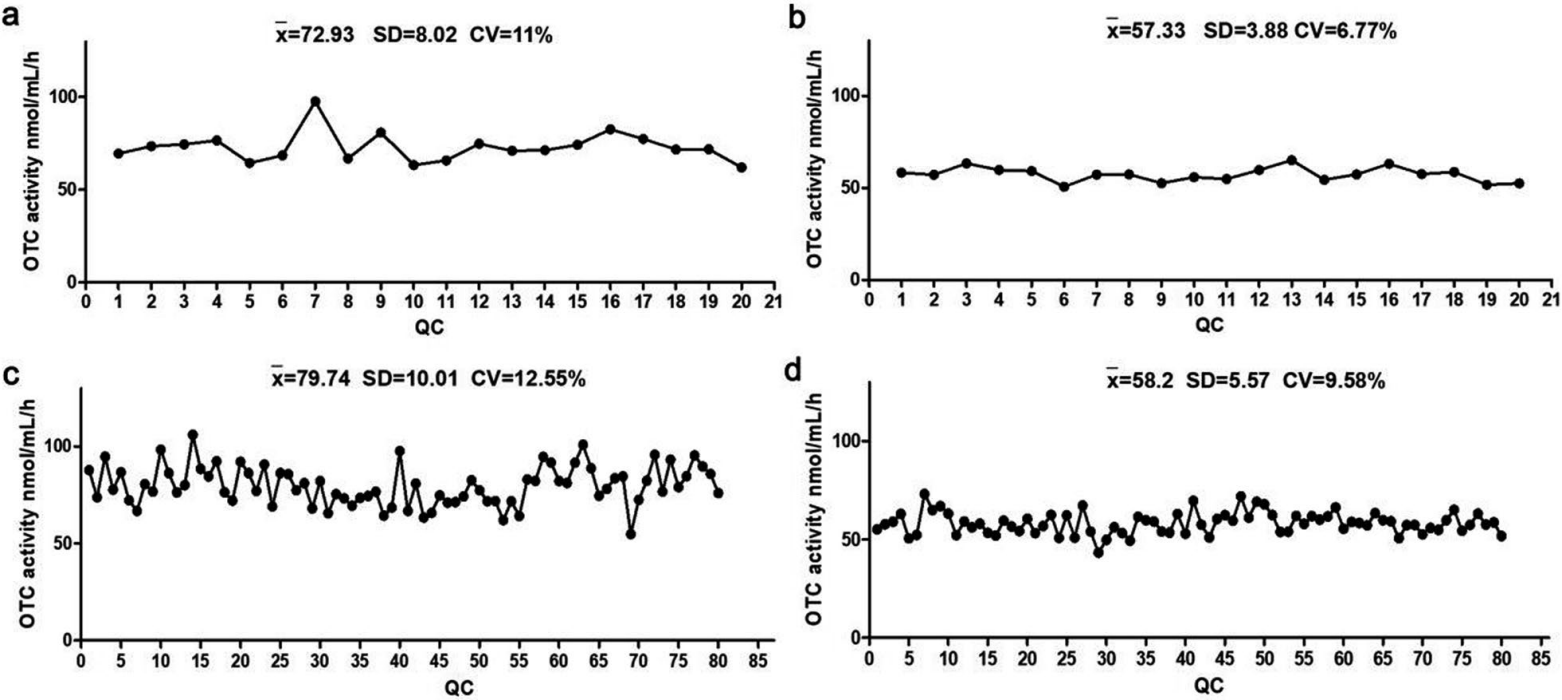
Imprecision evaluation of the OTC activity testing system. **a** The OCT activity detected after 1-h reaction time, n = 20. **b** The OCT activity detected after 3-h reaction time, n = 20. **c** The OCT activity detected after 1-h reaction time, repeated 4 times with 20 points each time. **d** The OCT activity detected after 3-h reaction time, repeated 4 times with 20 points each time

To advance the clinical viability of this detection system, we conducted a comprehensive assessment of its precision by evaluating intra-CV and inter-CV. Our findings revealed that the intra-CV for a 3-h reaction time (6.77%) was lower than that for a 1-h reaction time (11%) (Fig. 4a, 4b). Similarly, the inter-CV for the 3-h reaction time (9.58%) was decreased compared to the 1-h reaction time (12.55%) (Fig. 4c, 4d). No discrete values were found in either groups, indicating that longer reaction times improve the precision of this detection system.

#### Limit of blank

In this study, the blank sample was tested for 12 times, resulting in an average OTC activity of 0.28 nmol/mL/h and a SD of 0.17. The LOB, established at 0.56 nmol/mL/h, was calculated using the formula: LOB = mean + 1.645 * SD.

### The clinical application of OTC activity testing system

#### Reference interval

**Fig. 5:**
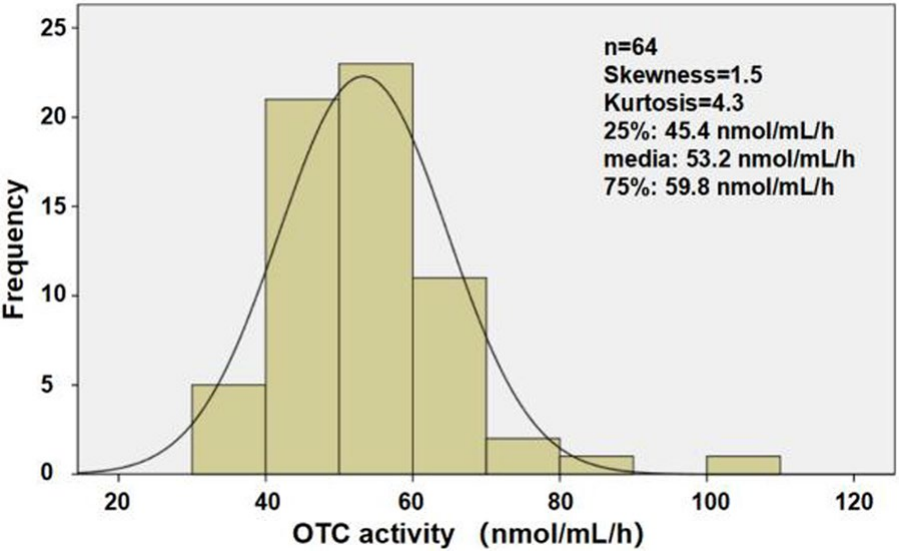
The frequency histogram of OTC activity in micro blood of normal neonates

To establish a reference range for the normal newborn population, we recruited 64 healthy newborns, regardless of gender, who had undergone genetic screening to exclude the impact of other Cit-related diseases. Initially, we conducted a normality test on the OTC activity in trace blood from these newborns. The frequency distribution chart revealed a skewed distribution in the normal population (Fig. 5), with a skewness of 1.5, and a kurtosis of 4.3, suggesting a notably positive skew. And interquartile range was 45.4 nmol/mL/h (25%) and 59.8 nmol/mL/h (75%), media was 53.2 nmol/mL/h.

For OTCD diganosis, the significance lies in the OTC enzyme activity falling below the reference range. Thus, We determined the reference value using a single-sided 95% confidence interval. Results showed the reference interval for the micro blood OTC activity in normal newborn population is greater than or equal to 39.6 nmol/mL/h.

#### Sensitivity, specificity and cutoff value

**Fig. 6:**
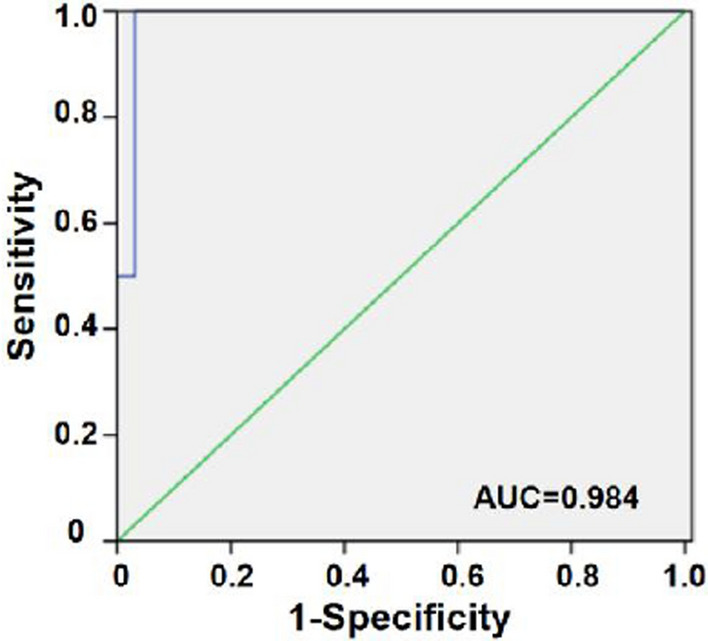
The clinical application of OTC activity testing system. The ROC curve of OTCD in normal newborns; NC = 64, OTCD = 2

To evaluate the diagnostic efficacy of this detection system for OTCD, we recruited 3 neonates with *OTC* gene mutations. Newborn 1 had a "Variant of Uncertain Significance (VUS)" mutation and exhibited low Cit level. Newborn 2 had a "Likely Pathogenic (LP)" mutation with abnormal level of Cit and plasma ammonia. Newborn 3 also had a "LP" mutation but showed no biochemical abnormalities. Currently, newborn 1 and 3 are healthy, while newborn 2 has passed away (Table 1). All of them declined liver biopsy and venous blood sampling that prompting us to detect OTC activity using micro blood. And results showed all three had low OTC activity (Table 1). Therefore, based on the diagnostic criteria, newborn 2 could be directly diagnosed with OTCD and newborn 1 could be diagnosed with OTCD after adding OTC activity detection. Although newborn 3 cannot be definitively diagnosed, we believe he is a suspected patient due to his gene positivity and low enzyme activity. Closer clinical follow-up is recommended. The ROC analysis indicated a sensitivity of 100% and a specificity of 96.9% for OTC activity detection (Fig. 6), reflecting strong diagnostic efficacy. Thus, incorporating OTC enzyme activity assays during the neonatal period can provide crucial diagnostic evidence and assist the diagnosis of positive patients and suspected patient.

**Table 1:** The clinical data of two neonatal OTCD patients

NO	Gender	Amino acid levels (μmol/L)			Plasma ammonia (μmol/L)	GC/MS (μmol/mmol Cr)		Mutations	Pathogenicity	Phenotype	OTC activity (nmol/mL/h)
		Cit	Glu	Gln		Orotic acid	Uracil				
1	male	5.24	ND	ND	ND	1.39	21.46	c.1015G > C	VUS	None	36.81
2	female	3.25	ND	ND	> 1000	ND	ND	c.484G > T	LP	Death	30.83
3	male	12.91	385.58	128.87	ND	2.38	22.05	c.385C > T	LP	None	27.46

*ND* Not detected. Normal reference interval: Cit, 6–23.6 μmol/L; Glu, 99.77–440.79 μmol/L; Gln: 275.38–937.03 μmol/L; Plasma ammonia: 18–72 μmol/L; Orotic acid: 0–6.33 μmol/mmol Cr; Uracil: 0–38.53 μmol/mmol Cr; the normal reference interval of OTC activity: ≥ 39.6 nmol/mL/h

## Discussion

Traditional biochemical strategies for diagnosing OTCD lack reliability in measuring Cit levels in the blood due to numerous influencing factors. More importantly, the advancement of newborn genetic screening is rapidly progressing, [27–29] and questionnaire surveys indicated that widespread support among residents for its implementation. [30] In the future, genetic screening for newborns will likely become standard clinic practice. And its widespread implementation means that more and more OTCD will be identified in the neonatal period, especially late-onset OTCD who were overlooked by traditional biochemical screening. [31] The clinical manifestations of OTCD exhibit substantial variability, which could stem from factors such as genetic inheritance, environmental influences, and specific variant sites within the individual's genome. [32] Currently, the classification of OTCD is heavily reliant on pre-existing reports. As a result, when dealing with newly discovered mutations or VUS, we are limited to follow-up monitoring. This constraint markedly hinders our capacity for precision in both diagnosis and treatment Grouping these patients and providing appropriate genetic counseling will become new challenges.

It is worth noting that, the residual enzymatic activity of OTC has been proved to reliably predict degree of this disease severity and time of onset based on cell-based expression system. [5] However, this in vitro detection system is time-consuming, labor-intensive, and lacks sufficient accuracy due to its detachment from the complex regulatory systems of the human body. It has been confirmed that OTC activity can be diagnosed through serum or plasma analysis. However, both methods require venous blood collection, which is challenging to obtain during the neonatal stage. As a result, this diagnostic approach has not yet been incorporated into newborn screening. Both biochemical and genetic screenings, integral though they are, exhibit intrinsic limitations. To address these shortcomings, we chose directly assess OTC activity in newborns' micro blood. We merged minimally invasive enzyme activity detected method into the initial recall process to promote the reliability of negative results within the population and to exclude other interfering factors, such as intestinal related diseases. Moreover, integrating enzyme activity testing into the diagnostic process can effectively shorten the duration of diagnosis and treatment. More importantly, this approach facilitates the diagnosis and estimation of the clinical phenotype of OTCD during the neonatal period and plays a crucial role in optimizing the OTCD neonatal screening strategy.

The methodology proposed here presents a novel approach to assessing OTC activity using micro blood from neonatal heel. We demonstrated strong linearity between OTC activity and enzyme concentration (R^2^ = 0.9793), reaction duration (R^2^ = 0.9922). Furthermore, intra-CV and inter-CV are crucial for methodological development. Lower intra-CV highlights the method's stability and reproducibility and lower inter-CV ensures consistency and stability across different times and experimental setups. [33] These factors are essential for establishing the robustness of the method. In this artical, the intra-CV and inter-CV were 11% and 12.5% with 1-h reaction time, respectively. Interesting, both values decreased to under 10% when extent the enzyme reaction time to 3 h. This improvement maybe attributed to the enzyme reaction stabilizing over a longer duration or to a more precise detection of higher product concentration by MS/MS. Vassef AA [34] reported the intra and inter-coefficient variation of colorimetry using serum ranged from 4.6%−19.2% to 5.1%−21.1%, respectively. Ishikawa H [35] found the intra-coefficient variation of colorimetry using serumwas 3.4–6.5%. Thus the method we established demonstrated a similar intra-coefficient variation and a lower inter-coefficient variation.

ROC analysis comprehensively assesses the diagnostic or predictive performance of a method by quantifying the trade-off between sensitivity and specificity, reflecting its ability to distinguish between true positive and true negative results by quantifying the trade-off between sensitivity and specificity. [36] A greater area under the ROC curve indicates stronger discriminatory capability and overall accuracy. The method we developed exhibited an area under the curve close to 1, signifying exceptional performance, coupled with remarkably high sensitivity (100%) and specificity (96.9%). Taiichi Wakiya [37] reported a sensitivity of 94.3% for the another colorimetric method which was lower than the sensitivity achieved in our study. It's more intriguing that, contrary to Jakub Krijt's findings [22], we didn’t detect significantly higher non-enzymatic Cit production in neonatal heel blood samples which indicating a potential discrepancy related to either the sample attributes or the methodology employed. This lower non-enzymatic production means it will not interfere with our testing system. Therefore, the method we employed is stable and repeatable.

From a clinical application perspective, Mutations in the *OTC* gene were identified in all 3 newborns, suggesting they were likely cases of OTCD. Among them, except for newborn 1, whose variant was classified as VUS, the others were deemed LP. Additionally, patient 1 had low Cit levels and OTC activity, but normal urine organic acid levels and is healthy currently. Newborn 2 had low Cit levels, low OTC activity, and elevated plasma ammonia, and unfortunately, she passed away. Newborn 3 had normal Cit levels, glutamine-to-glutamate ratios, and urine organic acid levels, but reduced OTC activity. Patients 3 is healthy by now. Therefore, after incorporating the micro-blood enzyme activity test, we are able to additionally diagnose neonate 1 as an OTCD patient and classify neonate 3 as a suspected case. In summary, we believe that OTC activity testing offers several key advantages: 1) enabling rapid diagnosis traditional biochemical screening shows low Cit levels, 2) aiding in the interpretation of VUS variant sites, and 3) distinguishing suspected patients in clinical settings when only genetic results are positive.

However, This article has a few limitations: 1) This method was not compared with established methods for OTC activity, 2) The lack of larger prospective research in this article, due to the low incidence rate of OTCD. And we anticipate supplementing relevant data in the future to address this gap, 3) The application of OTC activity measurement in dried blood spots is superior to the use of micro blood. It avoids the need to recall newborns and greatly reduces the false positive in primary screening. It is a pity that we have not yet established a method using dried blood spots that is sufficiently simple, stable and reproducible.

## Conclusion

In conclusion, our method utilizes a simpler approach by directly analyzing OTC activity using micro blood from neonatal heel. This method eliminates the need for venous blood collection, significantly enhancing compliance among parents of newborns in clinical practice. It success in identifying OTCD showcases its potential for streamlining and improving early detection strategies. By addressesing the limitations of existing screening strategies, this approach can be combined with emerging genetic screening to predict clinical phenotype, offering a promising alternative for rapid and efficient diagnosis.

## Data Availability

The experimental data and the simulation results that support the findings of this study are available in Figshare with the identifier https://doi.org/10.6084/m9.figshare.27686256.v1

## Abbreviations

OTCD: Ornithine transcarbamylase deficiency
OTC: Ornithine transcarbamylase
ORN: Ornithine
Cit: Citrulline
UCDs: Urea cycle disorder
CV: Coefficient variations
MS/MS: Tandem mass spectrometry
SD: Standard deviations
LOB: Limit of Blank
ROC: Receiver Operating Characteristic
VUS: Variant of Uncertain Significance
LP: Likely Pathogenic
